# Content and intracellular distribution of the inducing metal in the primary rhabdomyosarcomata induced in the rat by cobalt, nickel and cadmium.

**DOI:** 10.1038/bjc.1967.90

**Published:** 1967-12

**Authors:** J. C. Heath, M. Webb


### CONTENT AND INTRACELLULAR DISTRIBUTION OF THE INDUCING METAL IN THE PRIMARY RHABDOMYOSARCOMATA INDUCED IN THE RAT BY COBALT, NICKEL AND CADMIUM

J. C. HEATH AND M. WEBB

*From the Strangeways Research Laboratory, Cambridge*

Received for publication July 12, 1967

PREVIOUS studies in this laboratory have shown that cobalt, nickel and cadmium, when injected as the powdered metal into the thigh muscle of rats of the hooded strain, produce a high incidence of rhabdomyosarcomata, whereas a number of other metals, including iron, copper, zinc, manganese, beryllium and tungsten are not carcinogenic under these conditions (Heath, 1956, 1960; Heath and Daniel, 1964a, b; Heath, Daniel, Dingle and Webb, 1962). Tumours, which arise at any time from about 3 months after implantation of the active metals, and which metastasize readily in the primary animal, are transplantable into rats of the same strain. A cobalt-induced tumour, for example, has been maintained by transplantation for 13 years and is now in its 137th passage. Corresponding values for a nickel-induced and cadmium-induced tumour are $3\frac{1}{2}$ years and 54th transplant, and 6 years and 75th transplant respectively. All of these transplants preserve the characteristics of the primary rhabdomyosarcomata; in general the cobalt primary tumours are the least and the nickel tumours the most differentiated.

Since a metallic implant disappears from the site of injection, it is probable that the metal in the dissolved state is one of the main factors in the induction of malignancy. Ions of many of the above mentioned metals, whether carcinogenic or not, are inhibitory to tissue metabolism (e.g. Dingle, Heath, Webb and Daniel, 1962) and are toxic to chick and mammalian cells in culture (Heath, 1953; Daniel, 1961, 1964); no common specific biochemical activity of $Co^{2+}$, $Ni^{2+}$ and $Cd^{2+}$ has yet been demonstrated.

The work that is reported in this paper is designed to answer the following questions. Firstly, whether the tumours contain high local concentrations of the corresponding metallic ions; secondly, whether these ions are bound firmly and are retained after the dissolution of the metal implants, and, thirdly, whether the carcinogenic cations have a common pattern of distribution, and are associated primarily with a particular intracellular component or organelle.

### MATERIAL AND METHODS

*Biological methods.*—Primary tumours were induced by the injection of a suspension of the finely powdered metal (28 mg.; Johnson, Matthey & Co. Ltd., Hatton Garden, London, E.C.1.) in horse serum (0·4 ml.) into the thigh muscle of 2–3 month old rats of the hooded strain as described by Heath (1956) and Heath and Daniel (1964a, b). Tumours for biochemical work were taken usually between 4 and 5 months after the initial implantation. At this stage of development they were free from any significant central necrosis.

Transplants were maintained by serial passage of tissue fragments (about 1·5 mm.$^3$) from the edge of an actively growing tumour into fresh rats at intervals of about 3–4 weeks. Insertion of the tumour tissue into the thigh muscle was made with a large-bore hypodermic needle.

All experimental animals were fed *ad libitum* on Diet No. 86 (North-Eastern Agricultural Co-operative Society Ltd.) rat cubes, and had free access to water.

*Isolation of Cellular Components.*—All operations were done in a cold room at 4° C. The tissue was homogenized for 2 min. with 9 vol. 0·25 M sucrose in a modification (J. T. Dingle and C. Mallows, unpublished data) of the motor-driven homogenizer described by Aldridge, Emery and Street (1960). The homogenate was strained through nylon gauze (200 mesh) and fractionated by differential centrifugation, essentially by the method of Schneider and Hogeboom (1950). The nuclear and mitochondrial fractions were sedimented by centrifugation for 10 min. at 700 *g* and 10,000 *g* (Serval SS-3 centrifuge). Each fraction was washed by resuspension in 0·25 M sucrose followed by recentrifugation under the above conditions, the washings being combined with the supernatant material before the next step. The microsomes were recovered by centrifugation for 1 hr. at 105,000 *g* (Spinco Model L Centrifuge), the residual supernatant solution being termed the soluble fraction.

Additional preparations of cell nuclei only were made by the lissapol method of Gilbert and Radley (1964).

*Isolation of nucleic acids and deoxyribonucleohistone (DNP).*—DNA was isolated by the methods of Marko and Butler (1951) and Kirby (1957), and RNA by the methods of Kay and Dounce (1953) and Kirby (1956). Any modifications that were introduced into these procedures are mentioned in the text. DNP was isolated by extraction with 1 M NaCl, essentially as described by Mirsky and Pollister (1946).

*Determination of metal contents.*—Glassware was cleaned as described previously (Webb, 1949). Water was re-distilled in a pyrex glass still.

Portions (about 1–2 g. wet weight) of the tumour, or other tissue were digested in a mixture of conc. $HNO_3$ (17 ml.) and water (3 ml.) on a sand-bath at about 150°. The solutions were evaporated until almost dry residues were obtained. These were dissolved in, and made up to a suitable volume (usually 5 or 10 ml.) with 1 N HCl (prepared from the re-distilled, constant-boiling acid) and the solutions filtered before analysis. Little or no material remained insoluble in HCl after digestion with $HNO_3$, in contrast to either a $HNO_3$–$HClO_4$ mixture (Buell, 1939) or $H_2SO_4$.

In the analysis of subcellular components, most of which were isolated in 0·25 M sucrose, extraction of the metal ions was used whenever possible in preference to digestion. In this procedure, a suitable volume of each fraction was made 5% (w/v) with respect to trichloroacetic acid (TCA) and kept in a glass stoppered flask at 60° C. for several hours, usually overnight. The denatured protein was removed and washed with 5% (w/v) TCA, the combined extract and washings being adjusted to volume with TCA and 5 N HCl to give final concentrations of these acids of 5% and 1 N respectively. In control experiments in which portions of homogenates of primary cadmium, nickel and cobalt tumours were extracted with TCA and also digested with $HNO_3$, the results obtained by the two procedures were identical.

The concentrations of the metallic ions in digests and extracts were determined by atomic absorption with a Perkin–Elmer Model 303 Spectrophotometer. Excellent recovery of $Cd^{2+}$, $Ni^{2+}$ and $Co^{2+}$ was obtained when known amounts of these cations were added to homogenates of both rat liver and of the transplanted tumours before and after digestion, and there was no interference due to the high $Na^{+}$ contents of the solutions. TCA affected the relationships between absorption and concentration of the above cations to different extents and the standard reference solutions that were used in the analysis of extracts were prepared in 5% (w/v) TCA and 1 N HCl.

Most analyses were made either by direct reading, or by scale expansion in conjunction with the recorder-readout accessory. When concentration was necessary, the aqueous solution was adjusted to pH 3·0 in the presence of 0·2 vol. 0·1 M glycine-HCl buffer of this pH, and 0·2 vol. of an aqueous 1% (w/v) solution of ammonium pyrrolidine dithio-carbamate (K. & K. Laboratories Inc., Plainview, New York) were then added. The use of bromo-phenol blue as an internal indicator simplified the pH control and did not interfere with the subsequent analysis. After dilution as necessary to a volume twice that of the initial solution, the chelated cation was extracted into an appropriately smaller volume of methyl isobutylketone. As observed by others (e.g. Slavin, 1964; Sprague and Slavin, 1964a, b; Joyner and Finley, 1966) increased analytical sensitivity was obtained by the use of the organic solvent, and with a 4-fold concentration on extraction, it was possible to extend the lower limit of detection to one tenth of the normal value for $Co^{2+}$, $Ni^{2+}$ and $Cd^{2+}$. This procedure was particularly valuable in the recovery of cations from extracts that in addition to TCA contained variable concentrations of sucrose. In aqueous solutions the absorption due to a fixed concentration of any one of the above cations was decreased by increasing amounts of the sugar and analysis of each extract had to be made by the method of additions.

*Nucleic acid and protein determinations.*—DNA was determined by Burton's (1956) modification of the diphenylamine procedure, and RNA by the orcinol reaction (Mejbaum, 1939). Commercial preparations of salmon sperm DNA (California Corporation for Biochemical Research, Los Angeles, U.S.A.) and yeast RNA (British Drug Houses Ltd., Poole, Dorset) were used as standards. Protein was estimated by the colorimetric method of Lowry, Rosebrough, Farr and Randall (1951) with bovine serum albumin (Fraction V. Armour Pharmaceutical Co. Ltd., Eastbourne) as standard.

*Dry weights.*—These were determined on tissue samples dried to constant weight at 105° C.

### RESULTS

### *Contents of inducing metal cations in the primary tumours*

Analyses were made on tissues from a total of 50 primary tumours (22 induced by nickel, 16 by cobalt and 12 by cadmium). Most of the tumours, which ranged in weight from 4 g. to 25 g., were removed 16–20 weeks after implantation of the powdered metal. A few older tumours (39, 45, 49 and 53 weeks) also were included in the survey. Dry weight : wet weight ratios for the healthy tissue of the tumours varied from 0·147 to 0·183 (mean value 0·162) and there were no significant differences in this ratio for tumours induced by any one of the three metals.

The presence of the cation of the inducing metal was detected in peripheral tissue (i.e. the growing edge) of all tumours except a slow-growing nickel tumour that was analysed 53 weeks after the initial implantation. $Ni^{2+}$ was detected also in a lymph node tumour that developed in an animal in which nickel powder had been administered intramuscularly. This tumour was probably a metastasis that developed secondarily to the rhabdomyosarcoma, although primary tumours have been observed to arise in the lymph nodes in response to the deposition of nickel sulphide, transported from the site of implantation by macrophages (Daniel, 1966).

In general, the content of ions of the inducing metal decreased from the centre to the periphery, and also with the age of the primary tumour. In both primary nickel and cadmium tumours of similar age, the contents of the cations in the peripheral tissue were very variable (e.g. 0·4–22·4 $\mu$g. $Ni^{2+}$/g. wet wt.; 0·5–56·3 $\mu$g. $Cd^{2+}$/g. wet wt.), whereas in the primary cobalt tumours the $Co^{2+}$-concentration was somewhat more constant (0·35–3·8 $\mu$g. $Co^{2+}$/g. wet wt.). In some animals in which primary tumours were well developed at 16–20 weeks, significant amounts of the cation of the inducing metal (i.e. 0·5–2·0 $\mu$g./g. wet wt.) were found in muscular tissue from the opposite leg. The contents of the appropriate cation were also high in the liver, kidney and spleen, and in the limited number of analyses made, were always greater than in the peripheral tissue of the primary tumour. With cadmium implants in particular, very high amounts of $Cd^{2+}$ were found in these organs (Table I); for example, in one series of analyses, the $Cd^{2+}$ content of

TABLE I.—*$Cd^{2+}$ Contents of Peripheral Tissue from Primary Tumours Induced by the Intramuscular Implantation of Metallic Cadmium into Rats and of the Livers, Kidneys and Spleens of the Host Animals*

| | Rat number | | | |
|---|---|---|---|---|
| | 9761 | 9746 | 9523 | 9518 |
| | $Cd^{2+}$ content ($\mu$g./g. wet weight) | | | |
| Tissue | | | | |
| Tumour (peripheral tissue) . | 9·40 | 56·3 | 13·0 | 3·54 |
| Liver . . . . . | 348·0 | 123·0 | 137·1 | 404·0 |
| Kidney . . . . | 142·5 | — | — | 178·8 |
| Spleen . . . . . | 18·6 | — | — | 43·8 |

the liver was above 400 $\mu$g./g. wet wt., and was approximately double that of the kidney (179 $\mu$g./g. wet wt.), 9 $\times$ that of the spleen (43·8 $\mu$g./g. wet wt.) and more than 100 $\times$ that of the tissue from the primary tumour in the thigh (3·54 $\mu$g./g. wet wt.).

Under the conditions of the present analytical measurements, cations of the inducing metal were not detected in transplants of the cobalt and cadmium tumours, but $Ni^{2+}$ (0·45–0·55 $\mu$g./g. wet wt.) was found in some transplants of the nickel tumour.

*Intracellular distributions of cations of the inducing metal in primary tumours*

Although, in these studies, the tumours were dissected before homogenization, and any visible remnants of the original metallic implant were discarded, the presence of residual metal granules remained a potential source of error. Excessively high values for the cation contents of the initial homogenates, therefore, were considered to indicate contamination, and the results of analyses on the fractions of these preparations were rejected. In the representative results shown in

Table II the contents of $Co^{2+}$, $Ni^{2+}$ or $Cd^{2+}$ in the homogenates, with the exception of one preparation from pooled cobalt tumours, were within the limits of the analyses summarized in the preceding section. These results show a common pattern of distribution of ions of the inducing metal in all of the three types of primary tumour. Thus, only 15% or less of the total cation content was associated with the soluble fraction, and the remainder was bound by the sedimentable cellular components. The major part was recovered in the nuclear fraction, a smaller amount being found in the mitochondria, and little or none in the microsomes. The latter finding was unexpected, as it was thought that the foreign ions might replace part of the normal complement of $Mg^{2+}$, or other bivalent cation of the ribosome. Both $Zn^{2+}$ and $Fe^{2+}$ occur regularly in the ribosomes of liver and other tissues (e.g. Petermann, 1964), and were present in appreciable amounts (0·42 and 0·26 $\mu$g./mg. protein respectively) in the microsomal fraction of the primary cobalt tumour (Table II).

In each experiment of Table II the subcellular components were isolated from the same homogenate in order that the distribution of the cation of the inducing metal amongst the different fractions of the tumour tissue could be determined. With the exception of one series of $Cd^{2+}$ analyses, in which the recovery was low (88%) there was satisfactory agreement between the cation contents of the initial homogenates and the recovery in the subcellular fractions. It is well known, however, that these subcellular fractions, as isolated in 0·25 M sucrose, are not homogeneous, and the limitations of this method of fractionation have been summarized by Schneider (1961). In particular, the nuclear fraction is liable to contamination by intact cells, mitochondria and connective tissue fibres, and the disintegrated cell membranes also are known to sediment from sucrose homogenates with the nuclear fraction (e.g. Takenchi and Terayama, 1965). The results of Table II, however, show that contamination by unbroken cells and mitochondria cannot explain the high cation content of the nuclear fractions from the primary tumours. Furthermore, the close agreement that was obtained between the results of the four or more replicates of the nuclear suspensions that were analysed, is evidence for the absence of contamination of these fractions by either residual granules of the inducing metals, or by fibres of connective tissue that contained excessive amounts of bound cations. Significant redistribution of cations through adsorption on to the swollen agglutinated nuclei during the fractionation also seems unlikely from the results of a series of control experiments.

Thus, when low concentrations of $Co^{2+}$ and $Ni^{2+}$ were added respectively to homogenates of liver and transplants of the nickel tumour, before fractionation by differential centrifugation, both cations were bound by the sedimentable components (including the microsomes), but about 50% of each remained in the soluble fraction, and binding by the nuclei was not disproportionately great (Table III). An essentially similar distribution of $Co^{2+}$ was found in the subcellular components that were isolated from the livers of animals that had been injected with $CoCl_2$ (Table IV).

In other controls approximately equal weights of primary and transplanted cobalt and nickel tumours were homogenized and fractionated separately. The nuclear fractions from the transplants were then combined with the soluble fractions of the corresponding primary tumours, and portions of these suspensions analysed for the appropriate cations before and after removal of the nuclei by centrifugation. Binding of the cation by the nuclei under these conditions was

TABLE II.—*Intracellular Distribution of Cations of the Inducing Metals in Primary Cobalt, Nickel and Cadmium Tumours*

| Inducing metal | Content of inducing metal in initial homogenate: μg./g. wet weight original tissue | Content of inducing metal in initial homogenate: Total content (μg.) | Component | Fraction of homogenate: Nuclear | Fraction of homogenate: Mitochondrial | Fraction of homogenate: Microsomal | Fraction of homogenate: Soluble | Recovery of inducing metal in all fractions (% of total content of homogenate) |
|---|---|---|---|---|---|---|---|---|
| Nickel (Rat No. 8630, 8662, 8882, 8888 | 2·48 | 162·5 | Protein (mg.) | — | 212 | 111 | — | — |
| | | | $Ni^{2+}$ (μg.) | — | 25·7 | 2·2 | — | — |
| Nickel (Rat No. 9213–9219) | 22·05 | 1102·5 | Protein (mg.) | 1240 | 184 | 100 | | |
| | | | $Ni^{2+}$ (μg.) | 924·4 | 116·7 | 9·3 | 45·7 | 99·4 |
| Nickel (Rat No. 9514–9517) | 21·7 | 710·0 | Protein (mg.) | 1037 | 186 | 945† | | |
| | | | $Ni^{2+}$ (μg.) | 662·0 | 9·8 | 32·6† | | 99·2 |
| Cobalt (Rat No. 8316, 8323, 8650, 8652, 8871, 8876)* | 3·84 | 566·0 | Protein (mg.) | 5340 | 1087 | 585 | 4044 | |
| | | | $Co^{2+}$ (μg.) | 420·5 | 36·8 | 6·0 | 89·2 | 97·6 |
| Cobalt (Rat No. 9190, 9191) | 30·0 | 1245·0 | Protein (mg.) | 1440 | 403 | 161 | 1228 | |
| | | | $Co^{2+}$ (μg.) | 1167·2 | 71·1 | 0·0 | 51·1 | 103·5 |
| | | | $Zn^{2+}$ (μg.) | — | 67·1 | 68·3 | | |
| | | | $Fe^{2+}$ (μg.) | — | 46·1 | 41·6 | | |
| Cadmium (Rat No. 9224–9226) | 12·49 | 624·5 | Protein (mg.) | 1756 | 258 | 150 | 987 | |
| | | | $Cd^{2+}$ (μg.) | 438·6 | 16·3 | 0·0 | 95·5 | 88·4 |
| Cadmium (Rat No. 9752, 9760) | 3·01 | 162·5 | Protein (mg.) | 1832 | 364 | 1582† | | |
| | | | $Cd^{2+}$ (μg.) | 106·0 | 14·8 | 29·8† | | 92·6 |

* Random samples of the nuclear, mitochondrial and microsomal fractions of the homogenate of these tumours were examined by Dr. T. Hall of the Cavendish Laboratory, Cambridge, with the electron probe microscope. No contamination of any fraction with residual granules of the inducing metal was detected.

† In these analyses the microsomal and soluble fractions were not separated.

TABLE III.—*Distribution of $Co^{2+}$ and $Ni^{2+}$ amongst the Subcellular Components of Liver and the Transplanted Nickel Tumour after the Addition of these Cations to the Tissue Homogenates Before Fractionation*

| Homogenate | Cation added to homogenate | Cation concentration (µg./g. wet weight original tissue) | Cellular fraction | Total protein (mg.) | Total content of added cation (µg.) | Cation concentration (µg./g. protein) | Recovery % of added cation |
|---|---|---|---|---|---|---|---|
| Liver | $Co^{2+}$ (120 µg.) | 3 | Nuclear | 1420 | 21·0 | 14·8 | 91 |
| | | | Mitochondrial | 1763 | 33·2 | 18·8 | |
| | | | Microsomal | 224 | 7·1 | 31·7 | |
| | | | Soluble | 2704 | 48·1 | 17·8 | |
| Transplanted nickel tumour (Rat No. 9391, 9393) | $Ni^{2+}$ (675 µg.) | 15 | Nuclear | 1520 | 208·7 | 137·5 | 86 |
| | | | Mitochondrial | 650 | 63·4 | 97·6 | |
| | | | Microsomal | 242 | 46·0 | 190·0 | |
| | | | Soluble | 1927 | 345·8 | 179·6 | |

low, and accounted for 13% of the $Ni^{2+}$ (3·08 μg.) and 17% of the $Co^{2+}$ (8·1 μg.) in the soluble fractions.

TABLE IV.—*Distribution of $Co^{2+}$ Amongst the Cellular Components of Rat Liver After the Subcutaneous Injection of $CoCl_2$*

| Cellular fraction | Experiment I: Protein content mg. | Experiment I: $Co^{2+}$ content μg. | Experiment II: Protein content mg. | Experiment II: $Co^{2+}$ content μg. |
|---|---|---|---|---|
| Whole homogenate | — | — | — | 356·2 |
| Nuclear | 1550 | 57·8 | 1346 | 105·5 |
| Mitochondrial | 1622 | 35·7 | 1406 | 44·6 |
| Microsomal | 368 | 5·0 | 624 | 25·2 |
| Soluble | 2572 | 180·6 | 2424 | 158·4 |

In these experiments male rats (about 250 g. body weight) were injected subcutaneously with 0·5 ml. (≡1·25 mg. $Co^{2+}$) of an isotonic solution of $CoCl_2$ $6H_2O$ (42·5 mM) in aqueous NaCl (94·5 mM). After being fasted for 17 hr. the rats were killed and the subcellular fractions prepared from homogenates of the appropriately pooled livers.

To eliminate the possibility that metal-rich fragments of cell membranes contaminated the nuclear fraction and contributed to its high cation content, nuclei were isolated from each of the primary tumours by the method of Gilbert and Radley (1964), the purification of the preparations being followed by phase contrast microscopy. The use of the lissapol solution in this procedure seemed less liable to cause artifacts through displacement of bound cations than other aqueous media that contained either $Ca^{2+}$ (Schneider and Petermann, 1950; Wilbur and Anderson, 1951) or $Mg^{2+}$ (Widnell and Tata, 1964). Before analysis, the nuclei, which contained 17·5–21·0% DNA, were precipitated with ethanol (5 vol.) at 0° C. to remove the detergent, and the residue was then dried with ethanol and ether. The results of these determinations (Table V) confirmed that cations of the inducing metal were concentrated in the nuclei from each of the three primary tumours.

TABLE V.—*Contents of Cations of the Inducing Metals in the Isolated Nuclei of Primary Nickel, Cobalt and Cadmium Tumours*

| Primary tumour induced by | Cation content of tumour tissue: μg./g. wet weight tissue* | Cation content of tumour tissue: μg./g. dry weight† | Cation content of isolated nuclei μg./g. dry weight |
|---|---|---|---|
| Nickel | 27·0 | 166·7 | 1086·0 |
| Cadmium | 1·18 | 7·3 | 49·8 |
| Cobalt | 3·82 | 23·6 | 176·7 |

* These analyses were made on randomly selected fragments of the tumour tissue before homogenization.

† Calculated from the average dry weight : wet weight ratio of 0·162 (see text).

*Association of cations of the inducing metals with the nucleic acids of the primary tumours*

Both $Cd^{2+}$ (21–260 μg./g. DNP) and $Ni^{2+}$ (20–120 μg./g. DNP) were found in the deoxyribonucleohistone from primary cadmium and nickel tumours respectively. In similar preparations from three batches of the primary cobalt tumour, however, the $Co^{2+}$-content was too small to be determined accurately. Also this cation was not detected in the DNP from transplanted cobalt tumours that were removed 16–17 hr. after the subcutaneous injection of $CoCl_2$ into the hosts.

Most initial preparations of DNA were made by Kirby's (1957) method, with the exception that ethanol was used for precipitation. Thus, although treatment with RNAase was included in the purification of the DNA, the RNA-contents of the preparations were high (30–40%). The contents of the cation of the inducing metal in these mixed nucleic acids from primary nickel, cadmium and cobalt tumours ranged from 52–480, 50–260 and 0–100 $\mu$g./g. nucleic acid respectively. When ethoxyethanol and methoxyethanol were used in place of ethanol in the isolation procedure as described by Kirby (1957) the purity of the DNA preparations was increased to 92–95% and a lower, but consistent yield of 2·5–2·8 mg. DNA/g. wet weight tumour tissue was obtained. Only single preparations of DNA that were isolated in this way from the primary cobalt, cadmium and nickel tumours were analysed; these were found to contain 38 $\mu$g. $Co^{2+}$, 31 $\mu$g. $Ni^{2+}$ and 52 $\mu$g. $Cd^{2+}$/g. DNA.

One sample of crude DNA (327 $\mu$g. $Ni^{2+}$/g.; 66·5% DNA) that was isolated from a primary nickel tumour with ethanol as the precipitant, was refractionated with ethoxy- and methoxy-ethanol to yield a preparation that was 94·5% pure and contained only 7·8 $\mu$g. $Ni^{2+}$/g. The combined supernatant fractions were dialysed to remove the organic solvents and frozen dried to recover the RNA. This contained 1016 $\mu$g. $Ni^{2+}$/g. (98% of the $Ni^{2+}$ in the initial preparation) in contrast to the bulk RNA (yield 27 mg./g. wet weight tissue) that was isolated from a portion of the initial tumour and which contained 123 $\mu$g. $Ni^{2+}$/g.

These results indicate, therefore, that a significant portion of the total content of inducing cation that is associated with the nuclei of a given primary tumour is bound by the nucleic acid components, and, at least in the nickel tumour, binding by nuclear RNA is much greater than by DNA. Wacker and Vallee (1959), amongst others, however, have shown that the total heavy metal content of RNA is greater than that of DNA. Presumably, many of these cations are contaminants that are introduced during the isolation procedure, since the nucleic acids have a great capacity for base exchange (Kirby, 1957). The content of $Zn^{2+}$ in a number of the initial DNA preparations, for example, was found to vary widely (41–788 $\mu$g./g. nucleic acid) and was invariably less (41–73 $\mu$g. $Zn^{2+}$/g. nucleic acid) in those samples that were isolated by the detergent method. Thus, the method of isolation may lead to the redistribution of cations, particularly with the primary tumours on the denaturation of protein with phenol.

### DISCUSSION

It is known that intramuscular implants of cadmium, nickel and cobalt dissolve slowly in the tissue fluids. It is not unexpected, therefore, that in the tumours that are induced by these metals there is a decreasing concentration gradient of the corresponding cation from the centre to the periphery. The cation content also decreases with the age of the tumour as the metal is eliminated from the site of implantation. Transplants of these tumours, which develop in the absence of the inducing metals, neither concentrate nor require the corresponding cation in excess of any normal level for growth and survival.

Elimination of the implanted metals leads to the accumulation of the corresponding cations in the liver, kidney and spleen of the host. With cadmium implants the concentration of $Cd^{2+}$ in these organs is very high, and a study of the intracellular distribution of this cation in the liver for example, might be of interest. Although the histology of the liver and other metal-containing organs has not been investigated, macroscopically the tissues appear normal and thus an excessive content of ions of the carcinogenic metals alone seem insufficient to induce the malignant change. As suggested by Heath (1960) it is probable that the induction of tumours at the sites of implantation is due to the combined effects of mechanical and chemical damage by the abrasive particles of metal and the attempted regeneration in the presence of the toxic cation.

In each of the primary tumours most of the total cellular content of the inducing cation appears to be associated with the nuclear fraction. Smaller amounts are found in the mitochondria, 15% or less in the soluble fraction, and little or none in the microsomes. *In vitro*, free ribosomes are known to be precipitated by various bivalent cations which in lower concentrations, may inhibit amino acid incorporation into protein by the particles (Petermann, 1964). Possibly, the low uptake of $Co^{2+}$, $Cd^{2+}$ and $Ni^{2+}$ by the microsomes *in vivo* is related in some way to their high content of $Mg^{2+}$. The latter is known to antagonize the uptake of $Ni^{2+}$ and $Co^{2+}$ by both bacteria and mitochondria (M. Webb, unpublished results).

In most of the analyses that are summarized in Table II, the contents of $Co^{2+}$, $Cd^{2+}$ and $Ni^{2+}$ in the mitochondria of the primary tumours range from about 50–150 m$\mu$g. cation/mg. protein. By tracer methods Dingle *et al.* (1962) found that isolated rat liver mitochondria bound about 2 $\mu$g. $Co^{2+}$/mg. protein N when incubated in the presence of 0·5 mM $Co^{2+}$, a concentration that gave 75% inhibition of pyruvate oxidation. In published experiments, slightly higher values (2·6 $\mu$g. $Co^{2+}$ and 2·7 $\mu$g. $Ni^{2+}$/mg. protein N) have been obtained by atomic absorption analysis for the uptake of $Co^{2+}$ and $Ni^{2+}$ by rat liver mitochondria under the same conditions. Keto acid oxidation by mitochondria is extremely susceptible to inhibition by a number of bivalent cations; inhibition by $Co^{2+}$ for example is apparent at a concentration of 50 $\mu$M. Although figures are not available for $Co^{2+}$-binding by the mitochondria at this low concentration, it is probably of the same order as the values (33·8 and 176 m$\mu$g. $Co^{2+}$/mg. protein; i.e. about 200 m$\mu$g. and 1035 m$\mu$g. $Co^{2+}$/mg. protein N) that may be derived from the data of Table II. Thus, it is to be expected that the presence of the cations of the inducing metals in the tumour mitochondria will affect their oxidative metabolism. These considerations led to a separate study of the activities of the mitochondria from the primary and transplanted tumours, the results of which are summarized in the following paper (Daniel, Heath and Webb, 1967).

Both DNA and RNA from the primary rhabdomyosarcomata contain the cations of the inducing metals and, subject to the limitations that are mentioned in the Results section, it seems that at least part of the high cation content of the nuclei of each of these tumours is associated with the nucleic acid components. Recently, Menke and Sarif-Sarban (1966) have reported that $^{60}Co^{2+}$ is incorporated into the nucleic acids of the chick embryo, and that the binding of the cation by DNA reaches a saturation point at a dose of 10 $\mu$g. $Co^{2+}$/egg. For the four-times precipitated DNA the $Co^{2+}$ content (4 m$\mu$g./g. DNA) at this limit corresponds to average distribution of one cobalt atom in 46 $\times$ $10^6$ nucleotides. In the DNA preparations from the primary metal-induced tumours, the isolation of which involves dialysis and several precipitation steps, the cation contents are much larger than the value given by Menke and Sarif-Sarban (1966), but nevertheless are small when expressed as atoms/mole nucleotide. Studies on the properties of these nucleic acids are now in progress.

The results of Tables II and III show that the distribution of the inducing metal amongst sub-cellular fractions of the primary tumours differs quantitatively from that which occurs when the corresponding cations are added before fractionation to homogenates of transplanted tumours or liver. In particular, in the primary rhabdomyosarcomata 70–90% of the cellular content of the inducing metal is bound by the nuclei whereas in the latter systems binding by the nuclear fraction is much less (20–30%). Although these findings, coupled with those on the isolated nucleic acids, suggest that the carcinogenic metals interact with, and thus alter the genetic apparatus, it should be stressed that only well-developed tumours have been analysed, i.e. in which the malignant change had taken place many cell generations previously. Both $Zn^{2+}$ and $Co^{2+}$ are concentrated by spontaneous mammary tumours in C3H mice when the host animals are injected with the chlorides of these metals, and are found in preparations of DNP from these tumours (Heath and Liquier-Milward, 1950; 1951). It is possible, therefore, that ions of heavy metals interact more readily with the nucleic acids and nucleoproteins of any growing and dividing cells, malignant or normal.

### SUMMARY

Primary rhabdomyosarcomata that are induced in rats by the intramuscular implantation of powdered metallic cobalt, cadmium or nickel develop in the presence of excessive local concentrations of the dissolved metals. In each tumour the content of incorporated metal decreases from the centre to the periphery and also, as the implant dissolves and is eliminated from the site of implantation, with the age of the tumour. Transplants of these tumours do not either concentrate or need the corresponding cations in excess of any normal requirements for growth and survival.

In the different primary tumours the distribution of the three inducing metal ions is similar, but differs quantitatively from that found when these cations are added before fractionation to homogenates of either liver or the transplanted tumours. Most of the inducing metal that is incorporated intracellularly by the primary tumours is bound by the nuclear fraction. Smaller amounts are found in the mitochondrial and soluble fractions, and little or none in the microsomes. At least part of the high cation content of the nuclei is due to binding by the nucleic acids.

This work was done with the support of the British Empire Cancer Campaign for Research (J. C. Heath) and of the Medical Research Council (M. Webb).

### REFERENCES

ALDRIDGE, W. N., EMERY, R. C. AND STREET, B. W.—(1960) *Biochem. J.*, **77**, 326.
BUELL, M. V.—(1939) *J. biol. Chem.*, **130**, 357.
BURTON, K.—(1956) *Biochem. J.*, **62**, 315.
DANIEL, M. R.—(1961) *Rep. Br. Emp. Cancer Campn*, **39**, 337.—(1964) *Rep. Br. Emp. Cancer Campn*, **42**, 376.—(1966) *Br. J. Cancer*, **20**, 886.

DANIEL, M. R., HEATH, J. C. AND WEBB, M.—(1967) *Br. J. Cancer*, **21**, 780.
DINGLE, J. T., HEATH, J. C., WEBB, M. AND DANIEL, M. R.—(1962) *Biochim. biophys. Acta*, **65**, 34.
GILBERT, I. G. F. AND RADLEY, J. M.—(1964). *Biochim. Biophys. Acta*, **82**, 618.
HEATH, J. C.—(1953) *Rep. Br. Emp. Cancer Campn*, **31**, 240 and unpublished results.—(1956) *Br. J. Cancer*, **10**, 668.—(1960) *Br. J. Cancer*, **14**, 478.
HEATH, J. C. AND DANIEL, M. R.—(1964*a*) *Br. J. Cancer*, **18**, 124.—(1964*b*) *Br. J. Cancer*, **18**, 261.
HEATH, J. C., DANIEL, M. R., DINGLE, J. T. AND WEBB, M.—(1962) *Nature, Lond.*, **193**, 592.
HEATH, J. C. AND LIQUIER-MILWARD, J.—(1950) *Biochim. biophys. Acta*, **5**, 404.—(1951) *Rep. Br. Emp. Cancer Campn*, **29**, 176 and 185.
JOYNER, T. AND FINDLEY, J. S.—(1966) Atomic Absorption Newsletter. Perkin-Elmer Co., Norwalk, Connecticut, U.S.A., **5**, 4.
KAY, E. R. M. AND DOUNCE, A. L.—(1953) *J. Am. chem. Soc.*, **75**, 4041.
KIRBY, K. S.—(1956) *Biochem. J.*, **64**, 405.—(1957) *Biochem. J.*, **66**, 475.
LOWRY, O. H., ROSEBROUGH, N. J., FARR, A. L. AND RANDALL, R. J.—(1951) *J. biol. Chem.*, **193**, 265.
MARKO, A. M. AND BUTLER, G. C.—(1951) *J. biol. Chem.*, **190**, 165.
MEJBAUM, W.—(1939) *Z. physiol. Chem.*, **258**, 117.
MENKE, K. H. AND SARIF-SARBAN, M.—(1966) *Nature, Lond.*, **212**, 821.
MIRSKY, A. E. AND POLLISTER, A. W.—(1946) *J. gen. Physiol.*, **30**, 117.
PETERMANN, M. L.—(1964) ' The Physical and Chemical Properties of Ribosomes '. Amsterdam (Elsevier), p. 110.
SCHNEIDER, R. M. AND PETERMANN, M. L.—(1950) *Cancer Res.*, **10**, 751.
SCHNEIDER, W. C.—(1961) In ' Biochemists Handbook '. Edited by C. Long. London (E. & F. N. Spon Ltd.), p. 810.
SCHNEIDER, W. C. AND HOGEBOOM, G. H.—(1950) *J. biol. Chem.*, **183**, 123.
SLAVIN, W.—(1964) Atomic Absorption Newsletter. Perkin-Elmer, Co., Norwalk, Connecticut, U.S.A., **3**, 141.
SPRAGUE, S. AND SLAVIN, W.—(1964*a*) Atomic Absorption Newsletter. Perkin-Elmer Co., Norwalk, Connecticut, U.S.A., No. 17.—(1964*b*) Atomic Absorption Newsletter, Perkin-Elmer Co., Norwalk, Connecticut, U.S.A., No. 20.
TAKENCHI, M. AND TERAYAMA, H.—(1965) *Exp. Cell Research*, **40**, 32.
WACKER, W. E. C. AND VALLEE, B. L. (1959) *J. biol. Chem.*, **234**, 3257.
WEBB, M.—(1949) *J. gen. Microbiol.*, **3**, 418.
WIDNELL, C. C. AND TATA, J. R.—(1964) *Biochem. J.*, **92**, 313.
WILBUR, K. M. AND ANDERSON, N. G.—(1951) *Exp. Cell Research*, **2**, 47.


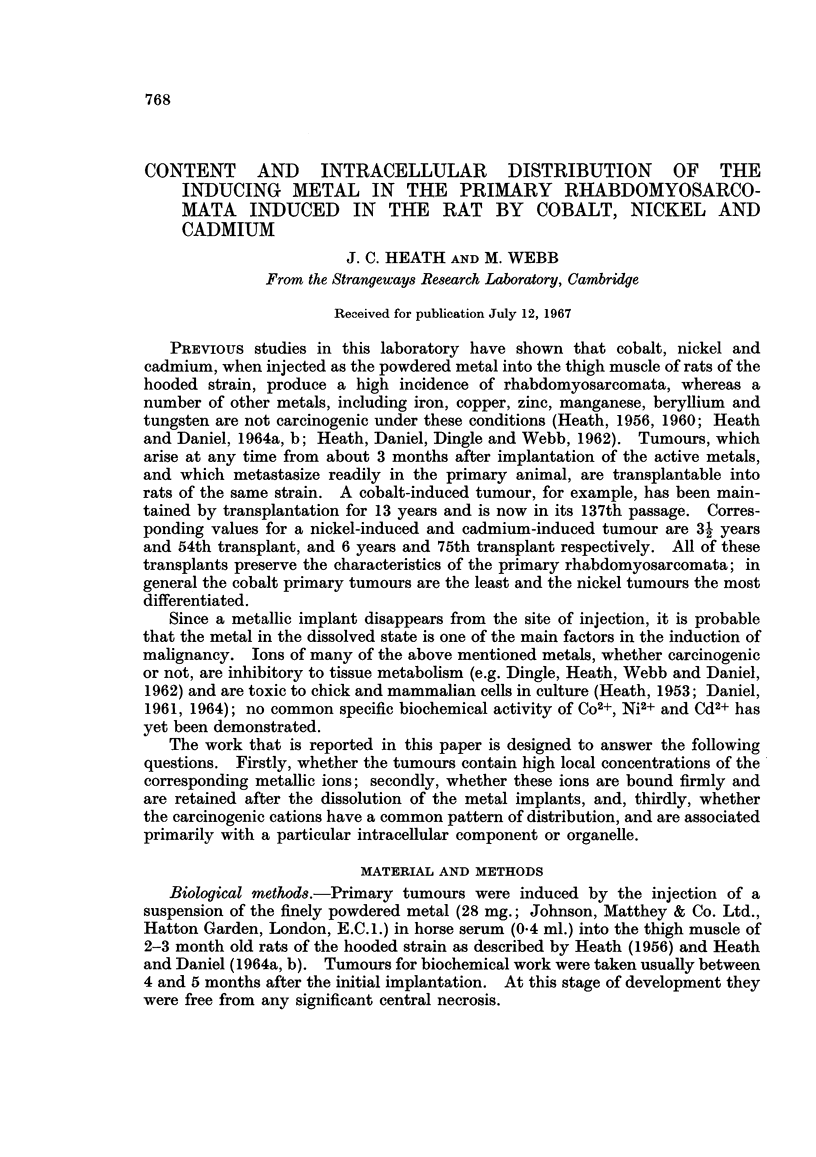

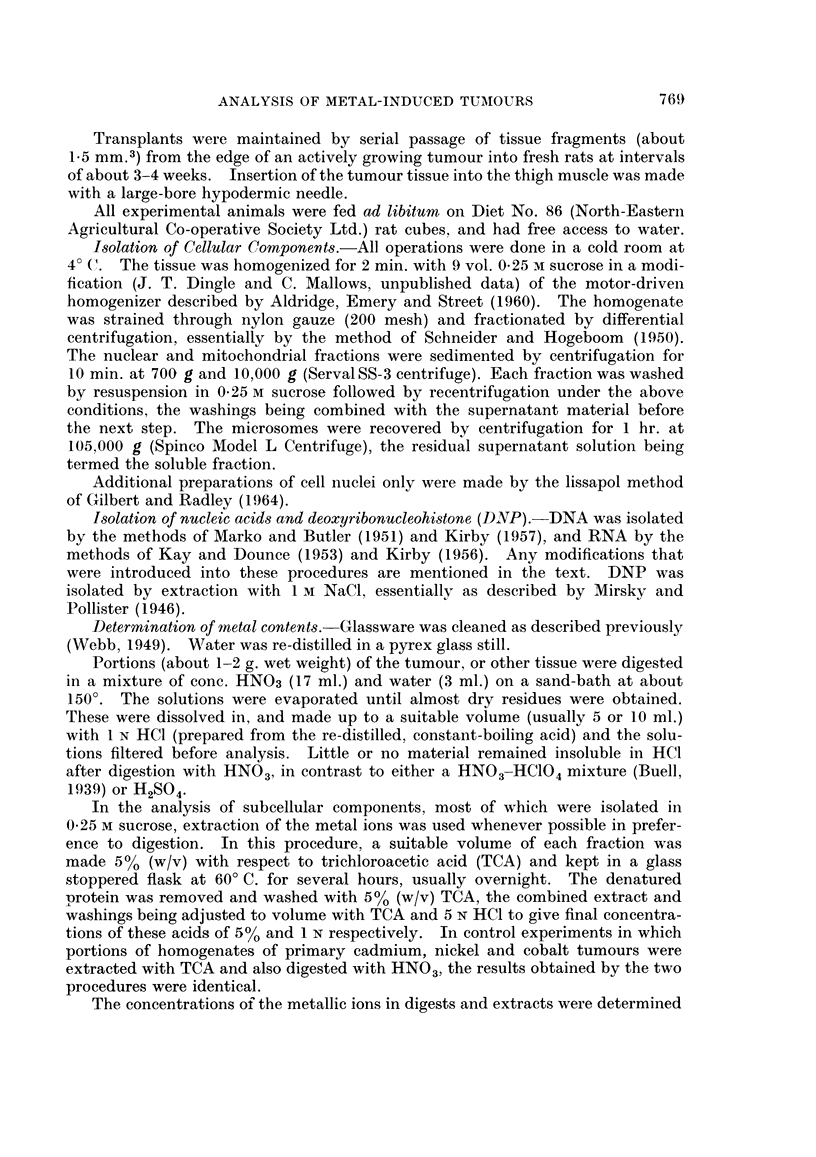

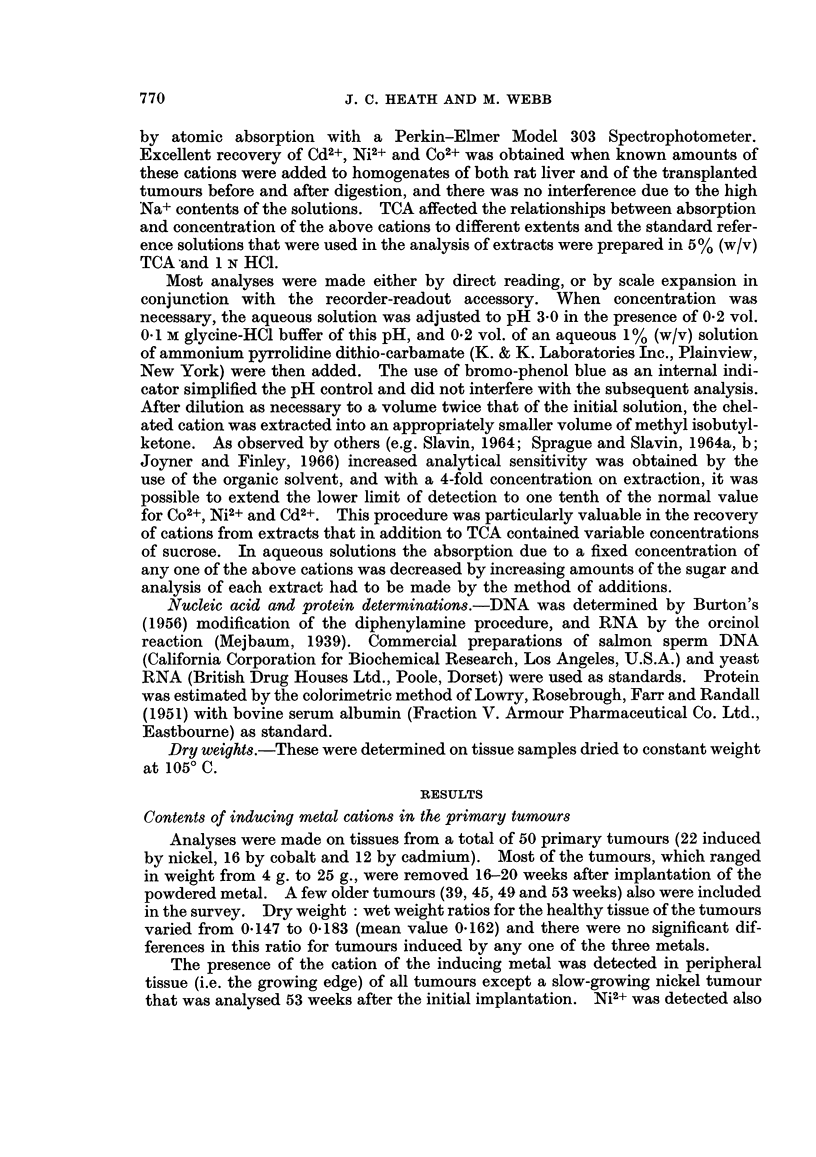

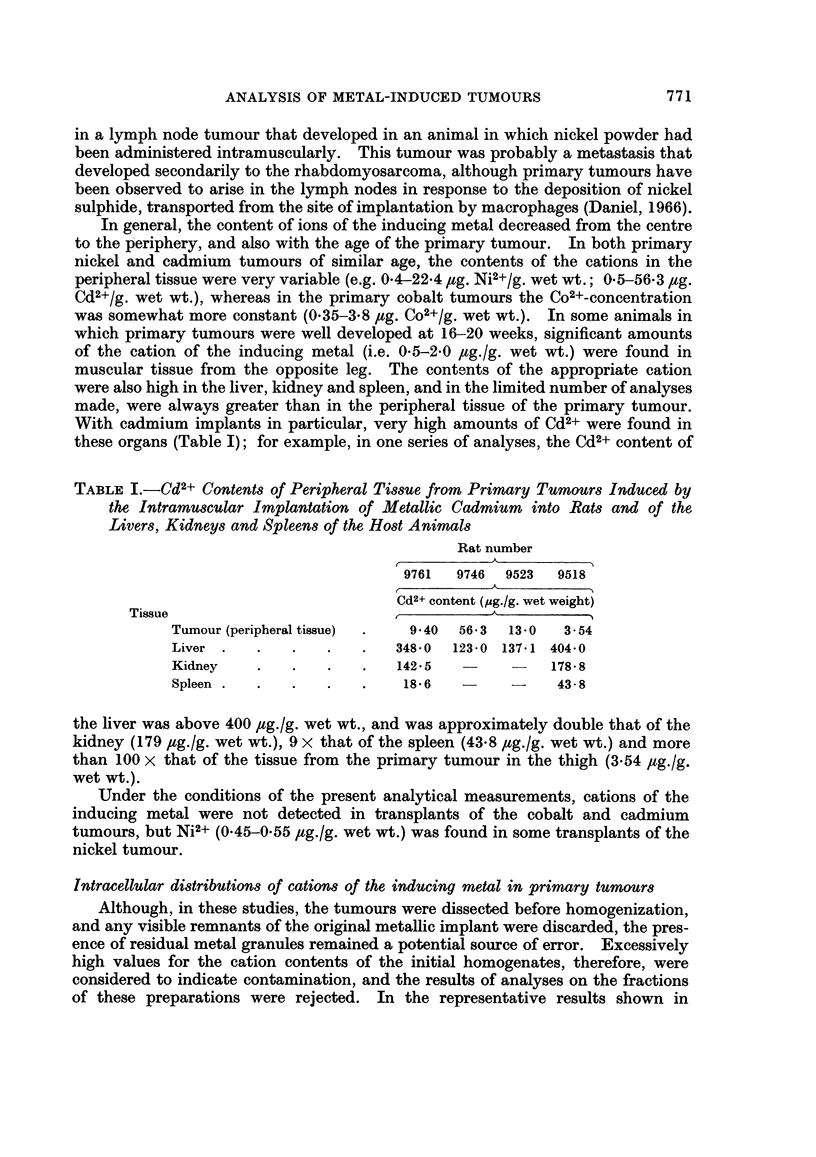

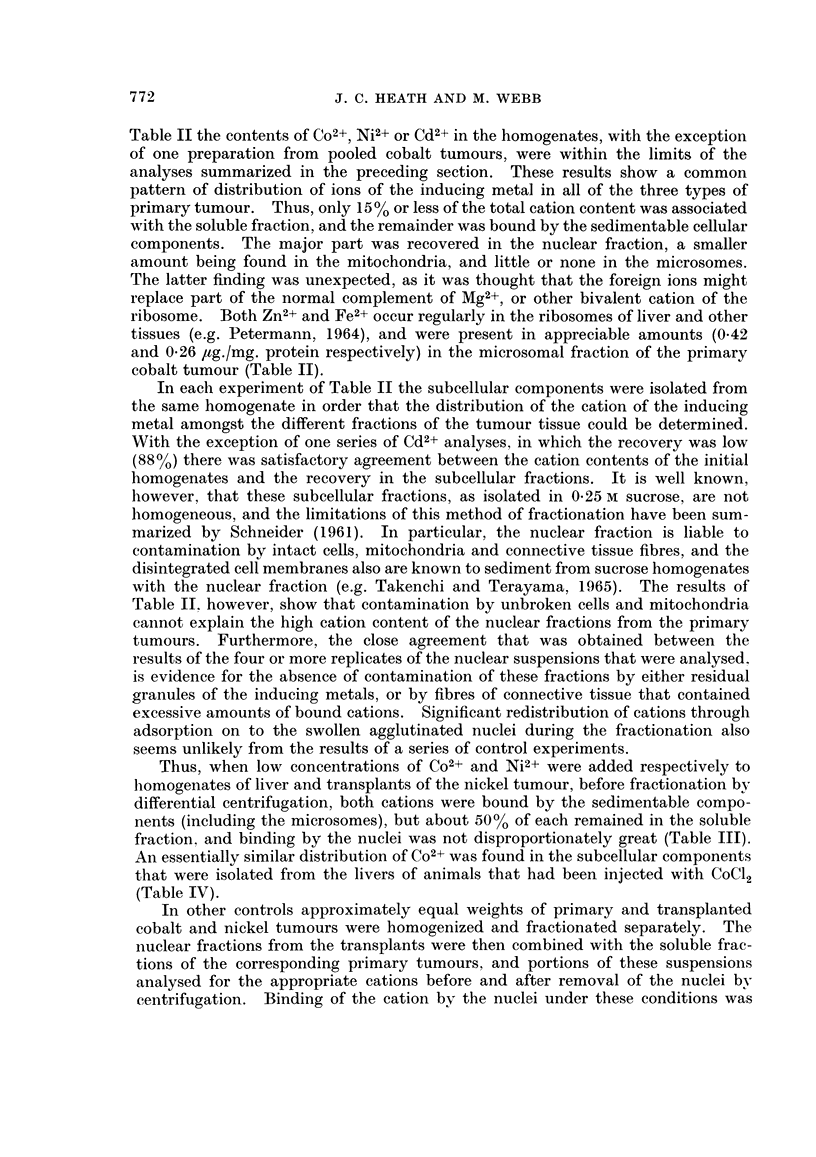

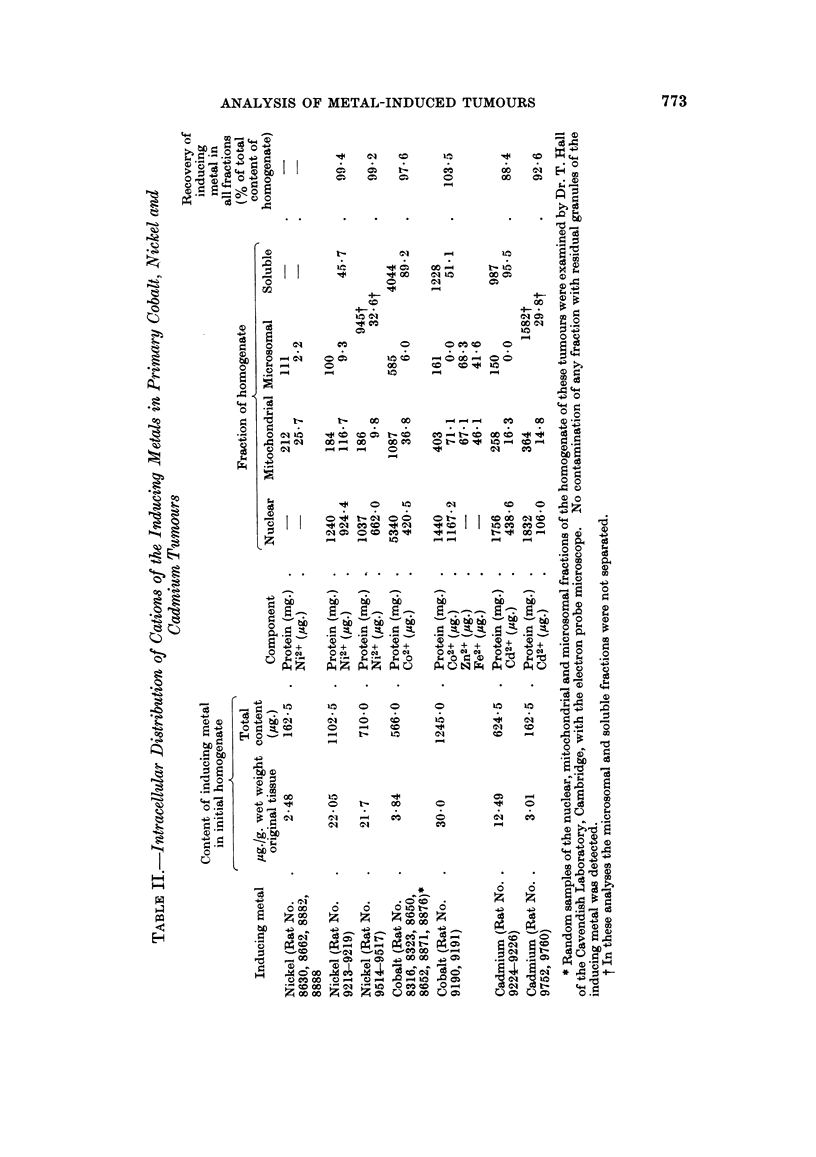

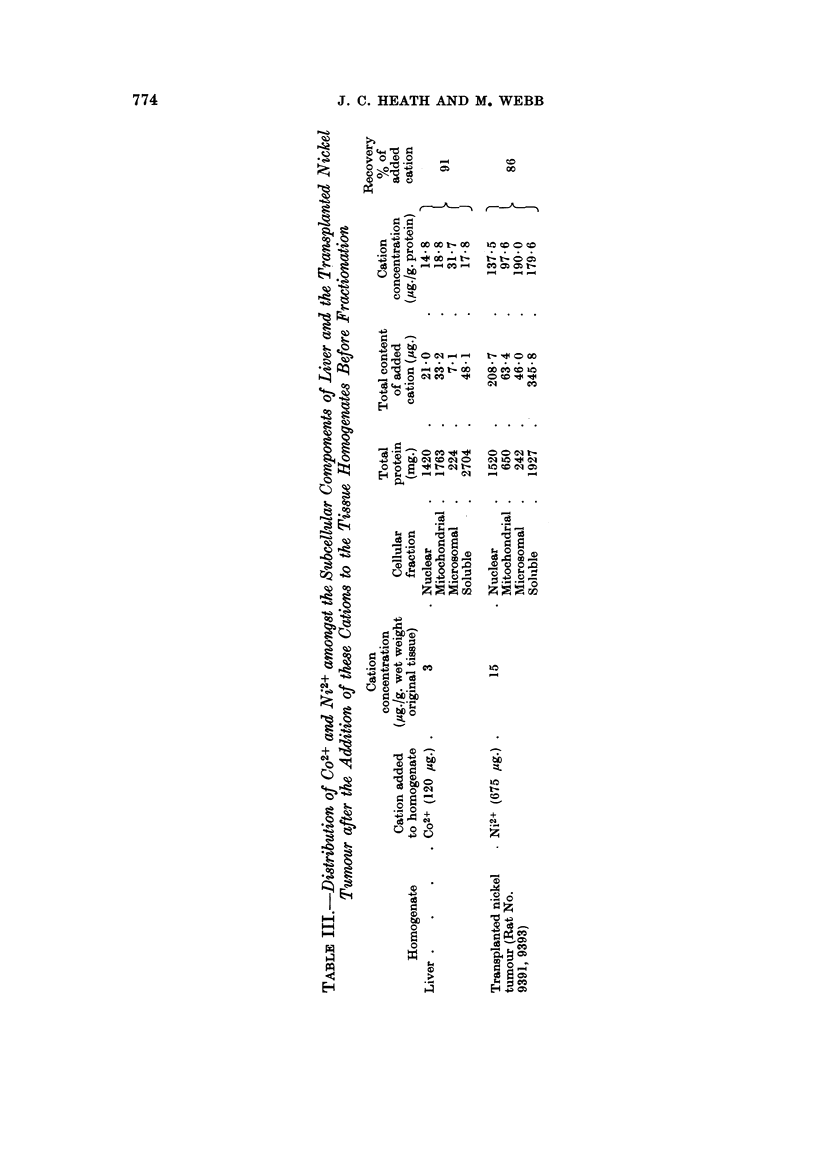

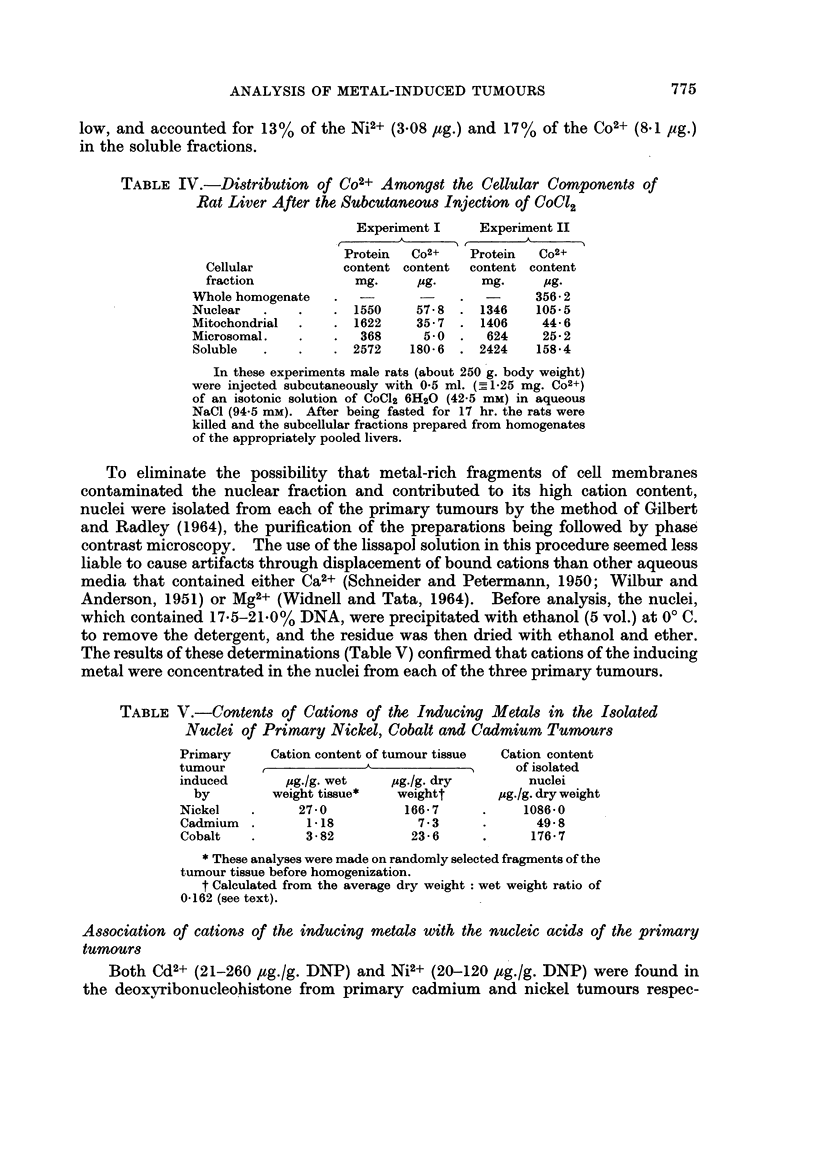

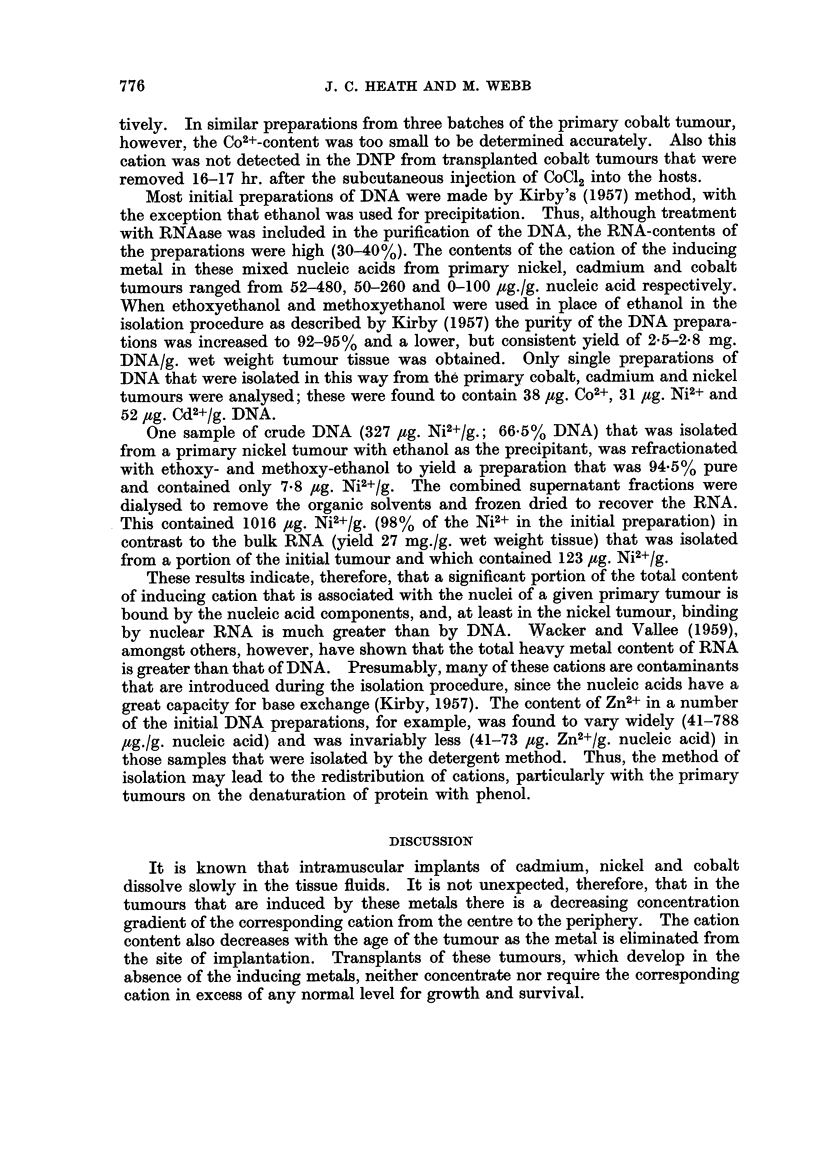

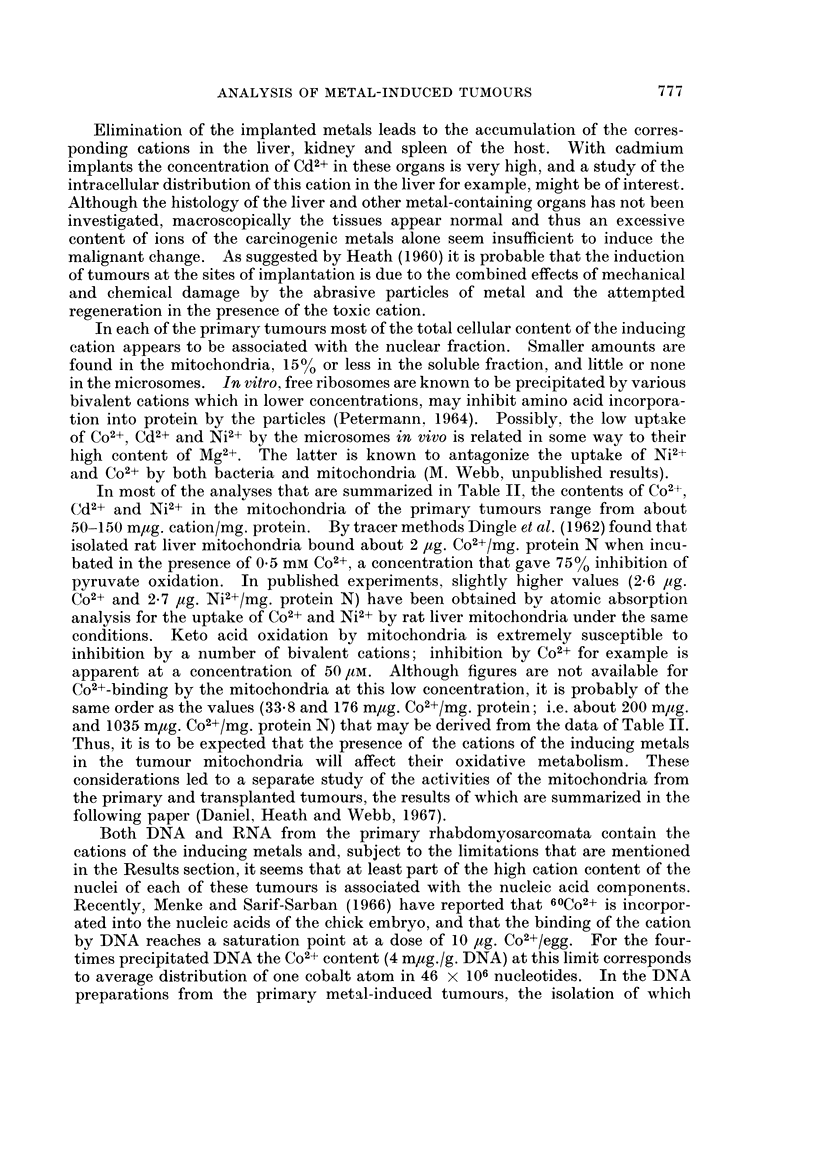

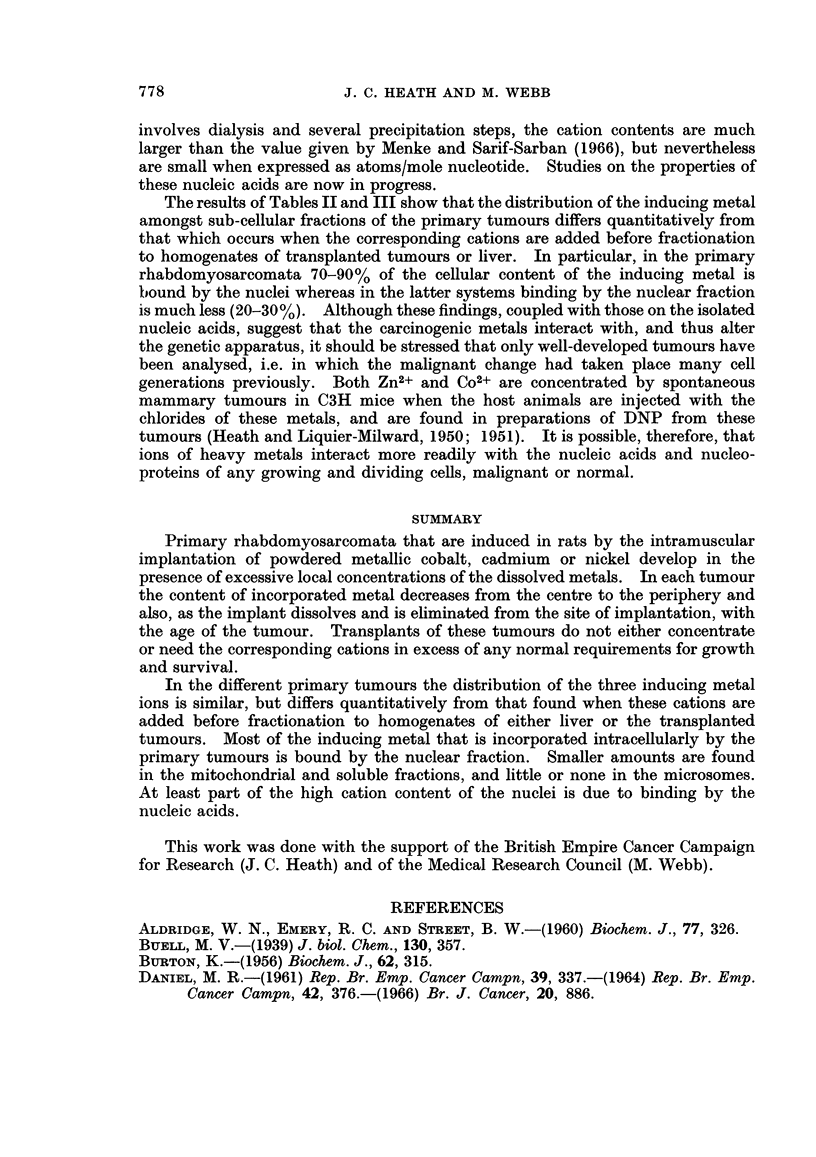

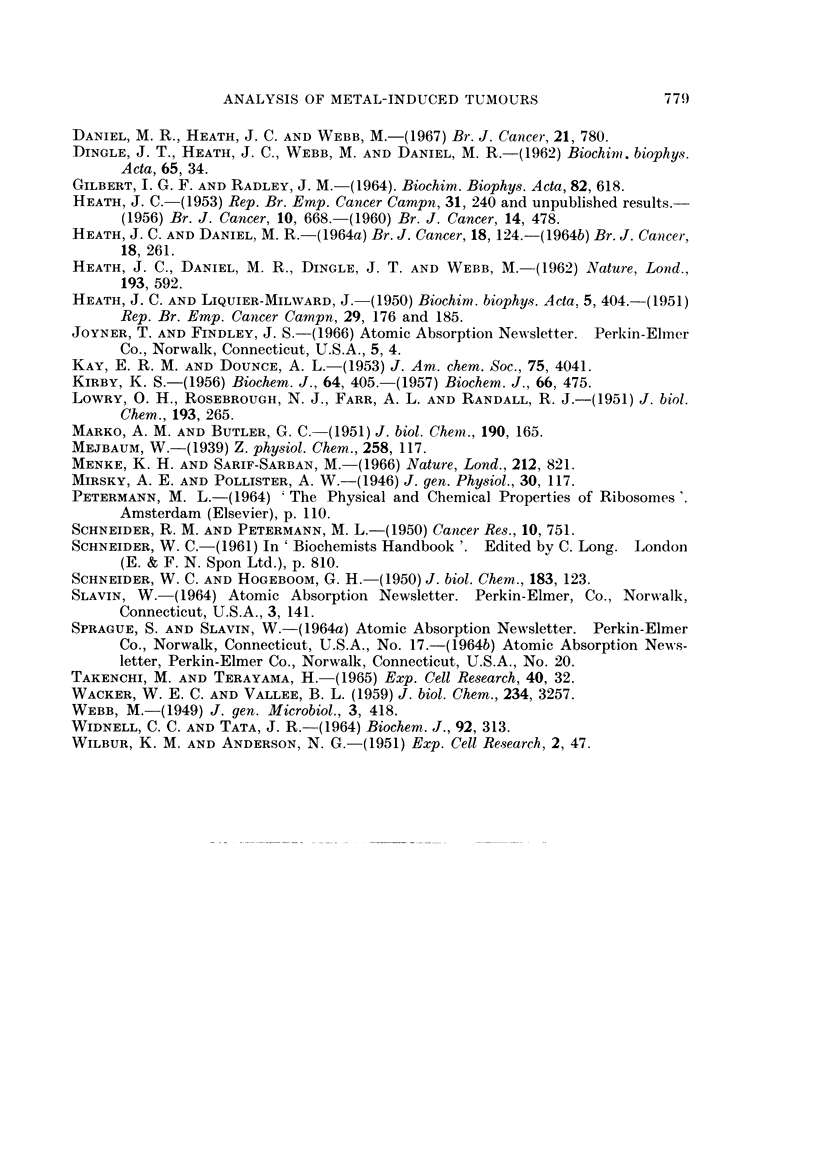


## References

[OCR_00775] ALDRIDGE W. N., EMERY R. C., STREET B. W. (1960). A tissue homogenizer.. Biochem J.

[OCR_00777] BURTON K. (1956). A study of the conditions and mechanism of the diphenylamine reaction for the colorimetric estimation of deoxyribonucleic acid.. Biochem J.

[OCR_00785] Daniel M. R., Heath J. C., Webb M. (1967). Respiration of metal induced rhabdomyosarcomata.. Br J Cancer.

[OCR_00791] GILBERT I. G., RADLEY J. M. (1964). A PROCEDURE FOR THE ISOLATION OF CELL NUCLEI FOR TRACE-METAL STUDIES.. Biochim Biophys Acta.

[OCR_00803] HEATH J. C., DANIEL M. R., DINGLE J. T., WEBB M. (1962). Cadmium as a carcinogen.. Nature.

[OCR_00797] HEATH J. C., DANIEL M. R. (1964). THE PRODUCTION OF MALIGNANT TUMOURS BY CADMIUM IN THE RAT.. Br J Cancer.

[OCR_00807] HEATH J. C., LIQUIER-MILWARD J. (1950). The distribution and function of zinc in normal and malignant tissues. I. Uptake and distribution of radioactive zinc, 65zn.. Biochim Biophys Acta.

[OCR_00814] KIRBY K. S. (1956). A new method for the isolation of ribonucleic acids from mammalian tissues.. Biochem J.

[OCR_00816] LOWRY O. H., ROSEBROUGH N. J., FARR A. L., RANDALL R. J. (1951). Protein measurement with the Folin phenol reagent.. J Biol Chem.

[OCR_00820] MARKO A. M., BUTLER G. C. (1951). The isolation of sodium desoxyribonucleate with sodium dodecyl sulfate.. J Biol Chem.

[OCR_00823] Menke K. H., Sarif-Sarban M. (1966). Incorporation of cobalt into nucleic acids of the chick embryo.. Nature.

[OCR_00830] SCHNEIDER R. M., PETERMANN M. L. (1950). Nuclei from normal and leukemic mouse spleen. I. The isolation of nuclei in neutral medium.. Cancer Res.

[OCR_00846] Takeuchi M., Terayama H. (1965). Preparation and chemical composition of rat liver cell membranes.. Exp Cell Res.

[OCR_00850] Widnell C. C., Tata J. R. (1964). A procedure for the isolation of enzymically active rat-liver nuclei.. Biochem J.

